# Development of Inspection Robots for Bridge Cables

**DOI:** 10.1155/2013/967508

**Published:** 2013-12-28

**Authors:** Hae-Bum Yun, Se-Hoon Kim, Liuliu Wu, Jong-Jae Lee

**Affiliations:** ^1^Department of Civil, Environmental and Construction Engineering, University of Central Florida, Orlando, FL 32816, USA; ^2^Department of Civil and Environmental Engineering, Sejong University, Seoul 143-747, Republic of Korea

## Abstract

This paper presents the bridge cable inspection robot developed in Korea. Two types of the cable inspection robots were developed for cable-suspension bridges and cable-stayed bridge. The design of the robot system and performance of the NDT techniques associated with the cable inspection robot are discussed. A review on recent advances in emerging robot-based inspection technologies for bridge cables and current bridge cable inspection methods is also presented.

## 1. Introduction

Bridges are important national assets that should be properly maintained to ensure public safety. Recent advances in bridge engineering have allowed bridges to be designed and constructed longer and slender than ever. In particular, long-span cable-supported bridges require high maintenance associated with reliable and efficient inspection methods.

Cable-supported bridges, including cable-suspension and cable-stayed bridges, consist of numerous subsystems, including pylons, anchorages, cables, stiffening girders, and slabs. A bridge cable system is an important subsystem that consists of main cables, hanger ropes, and stay cables. The cable rope is usually made of high-strength carbon steel that is five to ten times stronger than regular structural steels [1].

In maintenance of bridge cables, only few components are considered to be repairable, such as high-density polyethylene (HDPE) cable sheathings, neoprene boots, and elastomeric rings. In the presence of corrosion or fatigue damage in main tension elements (MTEs) near anchorages or in free spans, repairing damaged MTEs is practically impossible. Thus, preventative maintenance is vital to ensure the safety of cable systems. To accomplish this, the development of reliable inspection methods is imperative to assess material and structural conditions of cable systems [2].

In the United States, highway bridges should be visually inspected every two years [3]. Structural Health Monitoring (SHM) technologies have also been used for bridge condition assessment. Current bridge condition assessment methods, however, have some technical limitations. For example, near anchorages, cables at supports are often not visible since these cables are sealed with grout. In free spans, trolley and rolling devices are used to access cables, which is not the safest way to inspectors [4, 5]. Limitations of the current cable inspection methods can be overcome using emerging robotics technologies. The robotics technologies are usually combined with powerful nondestructive testing (NDT) techniques for bridge cable systems that are hardly accessible with current inspection practices. This paper reviews recent advances in robot-based inspection technologies for bridge cables. This paper also introduces a unique cable inspection robot that has been developed in Korea since 2010 as a part of the Super Long-Span Bridge R&D project led by Korea Ministry of Land, Infrastructure and Transport (MOLIT). Design of the cable robot system and performance test results of different NDT techniques associated with the robot will be presented.

This paper consists of two parts: (i) overview on current cable inspection methods (Sections 2 and 3) and (ii) introduction to the cable inspection robot developed in Korea (Section 4). In Section 2, an overview on current cable inspection methods will be described. In Section 3, commercially available cable-inspection systems will be described. Detailed description of the cable-inspection robot newly developed in Korea is presented in Section 5.

## 2. Current Practices for Bridge Cable Inspection

### 2.1. Trends in Construction and Maintenance of Cable-Supported Bridges

**Figure 1:**
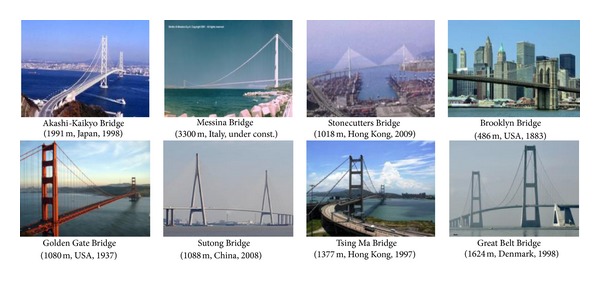
World-landmark cable bridges. The parenthesis shows the main span length, country, and constructed year.

An increasing number of cable-supported bridges are being constructed over the world, especially in the developing countries (see Figure 1). Construction technologies of cable-suspension and cable-stayed bridges were originally developed in the USA and Germany and then in Japan through the major construction projects of the Akashi-Kaikyo Bridge, a cable-suspension bridge, and the Tatara Bridge, a cable-stayed bridge. More recently, a number of cable-supported bridges have been constructed in China, including the Tsing Ma Bridge in 1997, the Sutong Bridge in 2008, and the Xihoumen Bridge in 2009. The construction of the Stonecutters Bridge is considered as a technological monument in bridge construction due to its unique design in composite towers and twin aerodynamic decks. The market size of cable-supported bridge construction is rapidly growing in Southeast Asian countries, including Thailand, Malaysia, Singapore, Vietnam, Philippines, Cambodia, and Bangladesh.

**Figure 2:**
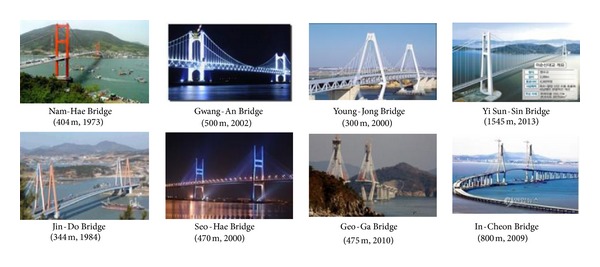
Cable-supported bridges in Korea. Numbers in parenthesis show the main span length and constructed year.

In Korea, the Nam-Hae Grand Bridge was constructed in 1973 as the first cable-suspension bridge, and the Jin-Do Grand Bridge was constructed in 1984 as the first cable-stayed bridge. The longest cable-suspension bridge in Korea is the Yi-Sun-Shin Bridge constructed in 2013, and the longest cable-stayed bridge in Korea is the Incheon Bridge constructed in 2010 (see Figure 2). Since Korean peninsula has many islands, the construction of long-span bridges is demanded to connect islands to the main land. Currently, a total of 64 bridges construction are planned in coastal areas. The market size of bridge construction is estimated over 1.2 billion U.S. dollars in next 20 years [7].

### 2.2. Current Maintenance Methods for Cable-Supported Bridges

#### 2.2.1. Maintenance of Cable-Supported Bridges

Reliable inspection methods are vital to ensure structural and service safety of cable-supported bridges. Bridge maintenance agencies employ different levels of inspection, including routine, periodic, emergent, and in-depth inspection.  Table 1 summarizes major bridge inspection manuals in the United States and Korea.

According to [8], bridges should be inspected every two years. In Korea, bridge agencies should perform regular inspection every 2 years and in-depth inspection every 5 years. In bridge inspection, it is important to observe excessive wear, broken wires, corrosion and pitting, state of lubrication, core condition, and so forth. Causes of defects and deteriorations must be understood using appropriate diagnosis methods. Frequent cable defects include surface rust, section loss, fatigue cracking, and collision damage.

#### 2.2.2. Cable Inspection Using NDT Methods

**Figure 3:**
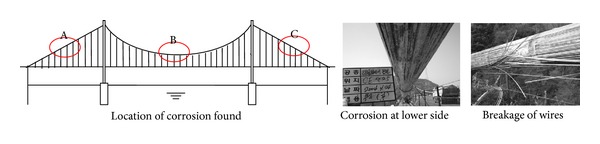
Inspection of the main suspension cables of the Nam-Hae Bridge [9].

Challenges in bridge cable inspection are mainly due to limited accessibility to cable systems. MTEs within cable bundles are often hardly visible to inspectors. Cables grouted in anchorage areas are very difficult to be inspected. Visual inspection and NDT methods for bridge cables in free spans are challenging due to inaccessibility of the cable. Early detection of internal damage is vital in preventive inspection. However, internal deterioration of bridge cables is hardly detectable using visual inspection methods. For example, Figure 3 illustrates the importance of early detection of internal defects. During the project of rewrapping main cables of the Nam-Hae Bridge in Korea, corrosion was found at the bottom of main cables at midspan and near anchorage zones of side spans. Breakage of wires was found at cable bands, which could cause serious structural failure if it was not found during the cable retrofit project. Some NDT methods, such as magnetic, ultrasonic, X-ray tests, have been used to detect such internal deterioration.

#### 2.2.3. Cable Condition Assessment Using SHM Methods

SHM technologies are widely employed to assess the structural conditions of cable bridges to ensure bridge safety and serviceability. The purposes of SHM are to diagnose and prognose a bridge's structural safety based on the measurement of structural behaviors. A SHM system consists of three subsystems, including sensing, data communication, and data processing. The sensing subsystem can continuously collect sensor data, such as service stresses, environmental stresses, and bridge deformation. The data communication subsystem transfers sensor data from a remote field-monitoring site to a data repository site and archives them. The data processing subsystem extracts meaningful information for bridge maintenance from raw sensor data.

A number of SHM systems have been employed for condition assessment of cable-supported bridges. An example is the Wind and Structural Health Monitoring System (WASHMS) for the Tsing Ma Bridge, Ting Kau Bridge, and Kap Shui Mun Bridge, developed by the Hong Kong Highways Department [10]. The sensing system consists of approximately a total of 900 sensors including accelerometers, strain gauges, displacement transducers, inclinometer, anemometers, temperature sensors, and dynamic weight-in-motion sensors: about 350 sensors on the Tsing Ma Bridge, 350 on Ting Kau Bridge, and 200 on Kap Shui Mun Bridge.

**Figure 4:**
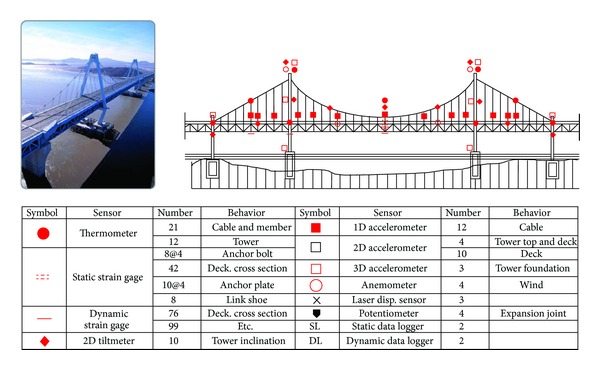
Structural health monitoring system of the Yeong-Jong Bridge in Korea.

In Korea, SHM technologies have been actively employed for cable-supported bridges.  Figure 4 shows the SHM system for the Yeong-Jong Bridge, which includes thermometers, strain gauges, tiltmeters, accelerometers, anemometers, displacement transducers, and potentiometers. Twelve accelerometers are installed throughout the bridge's cable system to monitor cable tension forces.

For condition assessment of bridge cables, measuring cable tension force is an important concern to assess structural safety. According to Tabatabai [2], although vibration-based force measurement is commonly used to estimate internal stress levels of bridge cables, the accuracy is arguable in many field applications. However, internal damage of cables, such as corrosion and inner wire breakage, can hardly be detected using the vibration-based method.

### 2.3. Cable Inspection Methods

Bridge cables are subject to various service and environmental stresses that could cause different modes of material and structural deterioration. Consequently, combinations of multiple NDT methods and instruments are used in cable inspection [12]. Current cable inspection methods are reviewed in this section.

#### 2.3.1. Visual Inspection

Human visual inspection is considered the primary cable inspection method over other NDT methods although human inspection relies heavily on subjective judgments of individual inspectors [2, 9]. In this method, first entire cable surface should be visually inspected from a close distance; then the inspection of neoprene boots, neoprene rings, visible guide pipes, and accessible anchorage surfaces is followed [2]. Visual inspections are often carried out using a cable inspection trolley that travels along cables at a low speed, which can be considered inconvenient and time consuming. Since the performance of an inspection trolley is largely affected by the existence of obstacles in cable paths, some part of cable systems, such as free cable spans with corrosion protection and anchorage areas, would be not accessible using this method [7].

#### 2.3.2. Image Processing-Based Inspection

Image processing-based cable inspection originates from a more general field of signal processing, including various image-processing techniques, such as image smoothing, image enhancing, segmentation, and edge detection [13]. Cable surface images are usually captured by a camera in a form of digital images as a two-dimensional numeric matrix. Image processing techniques are used to process these numeric matrices associated with pattern recognition algorithms to extract useful damage-related information [14]. Mandal and Atherton applied image-processing techniques to evaluate the severity of cable surface damage [15]. Ho et al. developed damage detection algorithms for cable inspection purposes [16]. These algorithms were designed to smooth and enhance the contrast of the original images and to classify damage patterns based on principal component analysis techniques.

#### 2.3.3. Vibration-Based Cable Force Measurement

Cable tension force can be calculated from vibration signatures of bridge cables using the following equation:

(1) $$\begin{array}{c} T=4L^{2}f^{2}m, \end{array}$$

where *T* is the cable tension; *L* is the cable length; *m* is the cable mass per unit; *f* is the natural frequency of the cable. Therefore, the cable tension force can be determined based on the measurements of those cable's physical properties [17]. This method, however, is not strictly applicable to bridge cable inspection due to oversimplification ignoring bending stiffness, sag under dead weight, and other complicating factors such as neoprene rings, viscous dampers, and variable stiffness along length.

Numerous vibration-based cable force measurement techniques have been developed. For example, Zui et al. developed a vibration-based method to measure cable forces, considering both the flexural rigidity and sag inherent to the inclined cable [18]. Cho et al. implemented Zui's method using wireless sensor networks [19].  Table 2 summarizes some accelerometer-based cable monitoring systems in different countries.

#### 2.3.4. Ultrasonic Inspection

Ultrasonic inspection techniques are also widely used in bridge cable inspection applications. An ultrasonic device consists of a transmitter sending high frequency sound waves through a specimen and a receiver to capture the reflected signal, while acoustic emission technique can detect breakage of a wire in a passive mode. Material defects as a discontinuity in a solid medium reflect the transmitted signal to a receiver as a signature of the presence of defects. Recently, long-range guided waves can be used as a transmitting signal [20]. Although the size and location of defects could be characterized based on the magnitude and delay time of the reflected signal [21], it requires calibrations through extensive laboratory experiments. The interpretation of ultrasonic test results could be subjective to the inspector's experiences and judgment.

Desimone et al. [22] conducted experimental study about the ultrasonic technique using a pulse wave for a wire with notches and grooves at different depth. Ultrasonic technique is applicable to inspecting cables connected to parallel wires in anchorage areas to detect wire fractures and corrosion [12]. This technique was applied to seven-wire cable strands at 12 anchorages of the Conhrane Bridge in Alabama, the United States [23, 24].

#### 2.3.5. Magnetic Methods

Magnetic sensors can be used to measure tensile stress in a cable, loss of metallic area (LMA), and local fault (LF), such as wire breakage. The mechanism of magnetic sensors is based on the sensitivity of a magnetic field to the presence of impairments, such as corrosion and fractures. Therefore, the change of a magnetic field along a cable indicates the presence of defects.

**Figure 5:**
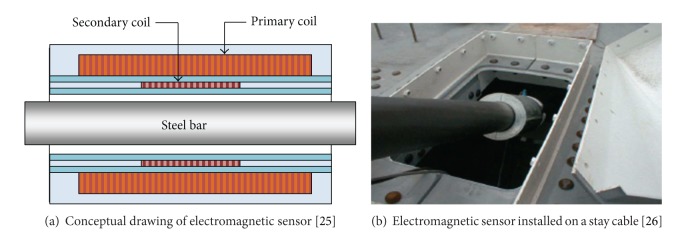
Electromagnetic sensors in bridge cable inspection applications.

A conceptual drawing of an electromagnetic (EM) sensor is shown in Figure 5. The EM sensor consists of a primary coil and a secondary coil to measure the apparent relative permeability and formalize EM characteristics of a steel rod (i.e., specimen). When a pulsed current flows in the primary coil, a ferromagnetic material is magnetized, and a pulsed magnetic field is introduced along the specimen. The relative permeability is a function of cable tension. The relative permeability (*μ*_*r*_) can be calculated as

(2) $$\begin{array}{c} \mu_{\gamma }=\frac{\mu_{0}\Delta B}{\Delta H}, \end{array}$$

where Δ*B* and Δ*H* are the change in the induction and magnetic fields, respectively, and *μ*_0_ is the permeability of the free space [25].

**Figure 6:**
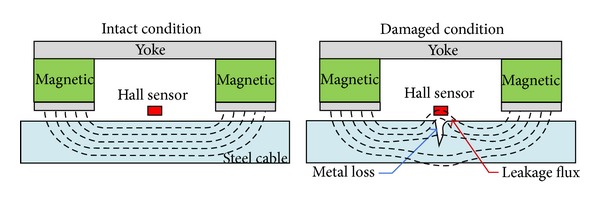
Mechanism of MFL-based damage detection [27].

Magnetic flux leakage (MFL) devices are often used to assess the severity of corrosion and detect local faults (LF) due to outer and inner wire fractures by measuring the loss of metallic cross-sectional area (LMA) [12]. The mechanism of MFL devices is illustrated in Figure 6. In an intact condition, the magnetic flux field generated using magnets remains uniform. In a damaged condition, metal loss due to wire corrosion or fractures disturbs the magnetic field, which results in inhomogeneous magnetic fluxes. The disturbance in the magnetic field can be detected using a Hall sensor placed between the poles of magnets. The magnitude of the Hall sensor signal is proportional to the magnetic flux leakage [15]. Therefore, the accuracy of LMA measurement is largely influenced with the homogeneity of magnetic flux around a steel cable. A strong permanent or an electromagnet is commonly used.

**Figure 7:**
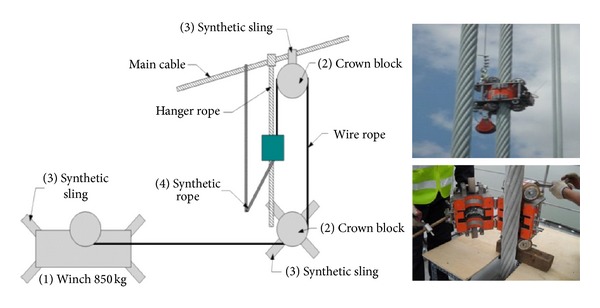
MFL system applied in hanger cable inspection for Yeong-Jong Bridge in Korea [32].

This technique is considered as a promising cable inspection method since LMA can be measured precisely under protective coating on cables nondestructively in relatively short time [12]. Typical applications of MFL devices include the steel cable inspection of aerial tramways, mining elevators, and offshore pipelines [13, 28, 29]. The MFL inspection technique also has been employed to different cable-stayed bridges [30, 31].  Figure 5(b) shows a MFL system applied in hanger cable inspection for the Yeong-Jong Bridge, Korea [32] (see Figure 7).

**Figure 8:**
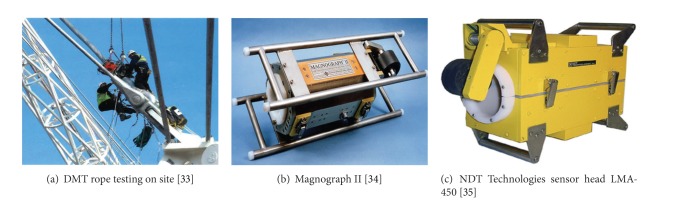
Commercially available magnetic sensor for bridge cable inspection.

There are a number of commercially available magnet sensors for steel wire ropes that can be used in bridge cable inspection. DMT GmbH developed magnetoinductive testing equipment for bridge cables (see Figure 8(a)). The equipment has strong permanent magnet heads for rope diameters of up to 150 mm. Brandt developed a rope testing head designed for inspection of steel cables with the diameter of up to 160 mm [37]. Kündig AG manufactures three PMK-series magnetic systems for wire rope diameters up to 125 mm [38]. Ropescan was developed by the British Coal Research Laboratory (now owned by Lloyds Beal Ltd.) to inspect mine-hoist locked coil wire ropes [39]. Canada Centre for Mineral and Energy Technology (CANMET) and the Noranda Technology Centre jointly developed Magnograph II, computer controlled wire rope testing equipment [34] (see Figure 8(b)). Magnograph can operate solely on Hall effect sensors which produce a signal independent of speed and are therefore able to operate at very low speeds. Intron Plus [32] and Laboratory LRM [40] manufacture testing heads that can inspect both flat and round steel wire ropes. These devices are typically applied in mine hoist ropes, on offshore platforms, cableways, cranes, lifts, and bridge cables [41].

NDT Technologies produces MFL magnetic sensor heads. The device can measure LF and LMA of wire ropes up to 120 mm in diameter. The device weight is less than 75 kg (Figure 8(c)). University of Stuttgart in Germany developed magnet sensors associated with an annular array of two sets of 30 Hall effect sensors for the applications of aerial ropeways, bridge suspension cables, ship lifts, and cranes [42].

#### 2.3.6. Radiography

**Figure 9:**
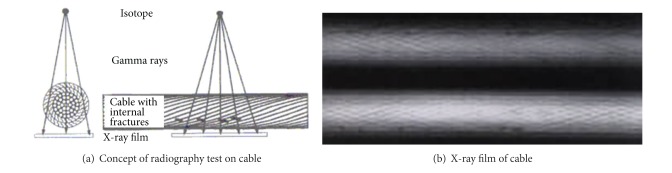
Radiography inspection for bridge cable inspection [36].

Radiography is used for subsurface imaging to detect cable defects using either X-rays or gamma rays. X-rays are produced using a high-voltage X-ray tube, and gamma rays are produced using a radioisotope (see Figure 9(a)). A summary of subsurface imaging technologies for reinforced concrete can be found in [43]. Radiography generally provides two-dimensional tomography for cross-sectional images of the three-dimensional object (see Figure 9(b)).

Xu et al. reported that the X-ray radiography can be applied to find cable defects in free spans, but not applicable for anchorages [48]. The application of radiography in bridge cable inspection is also limited due to possibility of radioactive hazards to working personnel while during inspection particularly at a high elevation of cable stay [1].

## 3. Cable Inspection Robots

Robotic systems can move along bridge cable systems, such as bridge cables, pipes, steel wires, and circular poles for repair and maintenance as well as inspection. This section introduces several robotic systems that can be used for the inspection of bridge cable systems.

### 3.1. Pipe Inspection Robots

**Figure 10:**
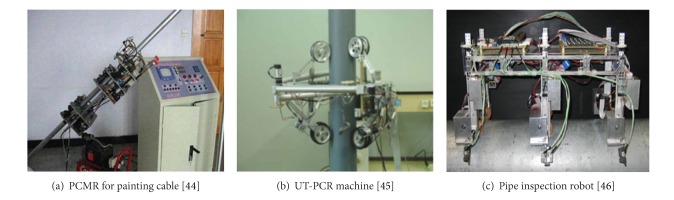
Pole and pipe inspection robots.

Li et al. developed a cable pneumatic climbing maintenance robot (PCMR) for coating and painting pipe structures (see Figure 10(a)) [44]. A pole climbing robot (UT-PCR) was developed by the University of Tehran, Iran, with pole grasping and vertical movement capability (see Figure 10(b)) [45]. The robot consists of a trilateral-symmetric body in a triangular shape with six limbs connected at its corner points through separate extension springs. The mechanical limbs are employed to grasp a pole. The wheels on the lower limbs are actuated by DC motors, while the wheels on the top limbs only guide the robot's movement. The robot has a self-locking mechanism to prevent sliding down on a pole.

A pipe-surface inspection robot was developed by Akita Prefectural University in Japan for pipe inspection (Figure 10(c)). This robot can traverse flanges, climb vertical flanges, and move along the bottom side of a pipe using six magnetic leg wheels.

A pole climbing and manipulating robot, developed by Shariff University, Iran, can pass bends and branches of a pole [49]. The robot consists of three main body parts, including 3-DOF planar substructure, *z*-axis rotating substructure, and grippers. The robot is actuated by three DC motors controlled by a central computer through control drivers.

### 3.2. Cable Inspection Robots

**Figure 11:**
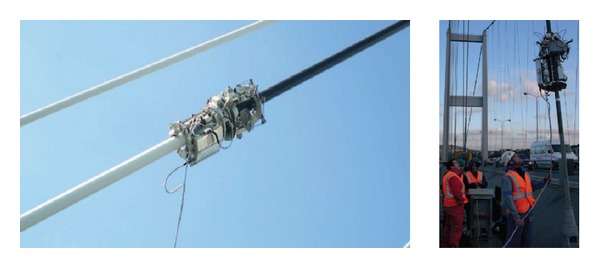
ATIS cable robot developed by Alpin Technik Leipzig [47].

ATIS Cable Robot, a cable-climbing robot developed by Alpin Technik Leipzig, was designed to mount different modules to meet various purposes of cable inspection and maintenance, including visual inspection and MFL modules for inspection and taping and welding modules for maintenance (see Figure 11). The climbing robot is designed for different cable diameters from 24 mm to 350 mm. A relatively short installation time of 5 to 15 minutes is an advantage of this system.

**Figure 12:**
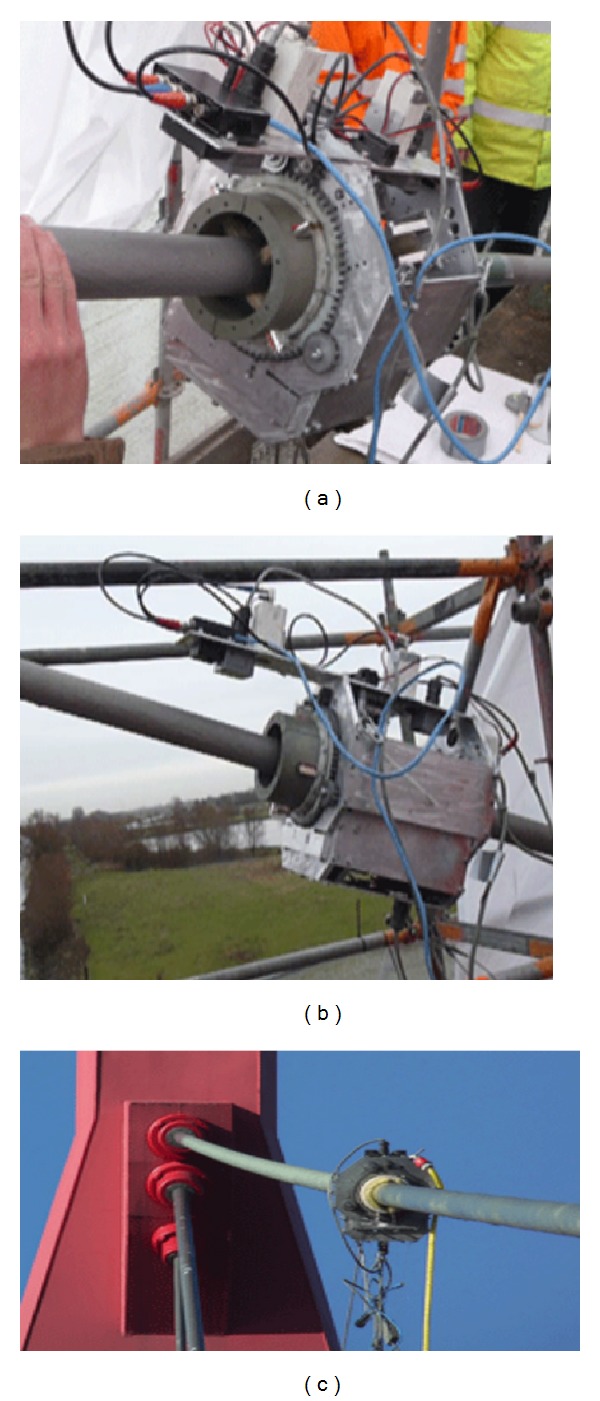
Mavis ReCreator and visual inspection [51].

Mavis ReCreator is another cable climbing robot developed by Tiefenbach GmbH (see Figure 12). This robot has five modules for cable coating maintenance, including coating removal, cleaning, repair, coating, and moving. Visual inspection instruments can be also mounted to the robot for surface inspection of bridge cables.

**Figure 13:**
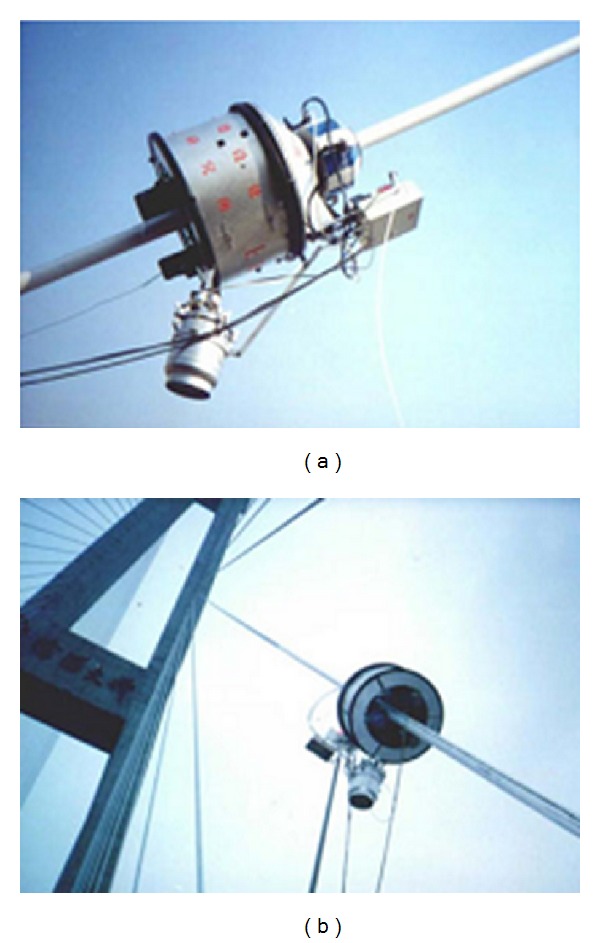
In situ painting experiment of cable maintenance robot [50].

Luo et al. (2005) developed a robotic system that can inspect, clean, and paint bridge cables (see Figure 13) [50]. The inner frame connected to the climbing module can move along the cable in spiral motion. The outer frame connected to the maintenance module balances the robot while it moves controlling hanging weights of the painting bucket and battery.

A mobile robotic system for inspection of power transmission lines, developed by Tokyo Electric Power and Toshiba, consists of vehicle assembly, guide rail, guide rail manipulator assembly, and balancer [52]. The robot can navigate a ground wire and maneuver over obstacles on ground wires. When the robot encounters a tower, a foldable arc-shaped arm that acts as a guide rail is unfolded to attach the arm to a ground wire placed on the opposite side of the tower.

## 4. Development of Cable Inspection Robot in Korea

**Figure 14:**
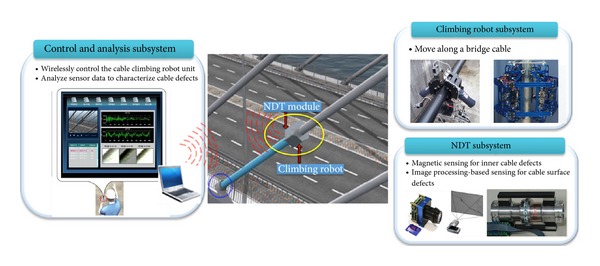
System components of cable inspection robot developed in Korea.

A research initiative has been supported by Korea Ministry of Land, Transportation, and Maritime Affairs (MLTM) since 2010 to develop a bridge cable inspection robot system. Although there are many commercially available systems as addressed in the previous chapters, challenges still remain to improve the performance of the inspection robot system (e.g., inspection speed, physical size, adaptability, controllability, etc.). The robotic system consists of three main subsystems, including (i) the climbing robot subsystem, (ii) the NDT subsystem, and (iii) the control and analysis subsystem (see Figure 14).

**Figure 15:**
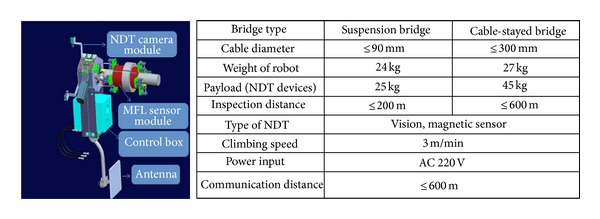
Design and specifications of cable inspection robot unit with the climbing robot subsystem and NDT subsystem.

The climbing robot subsystem allows the robot unit to move on a bridge cable. The robot is controlled wirelessly by inspectors and can transmit sensor data collected from the sensing modules to the control and analysis subsystem. The NDT subsystem consists of two sensing modules, including (i) the magnetic sensing module using MFL devices to detect inner wire defects, such as LF due to wire fractures and LMA due to wire corrosion, and (ii) the image processing-based sensing module to detect defects on cable surface.  Figure 15 shows the design and specifications of the climbing robot subsystem and NDT subsystem. Two types of the climbing robot system were developed for cable-suspension bridges and cable-stayed bridges. This is necessary since cable-stayed bridges have inclined cables with larger gauges (up to 300 mm), and cable-suspension bridges have vertical hanger cables with smaller gauges (up to 90 mm). Detailed description of each subsystem will be presented in the subsequent subsections.

### 4.1. Climbing Robot and Control Subsystems

**Figure 16:**
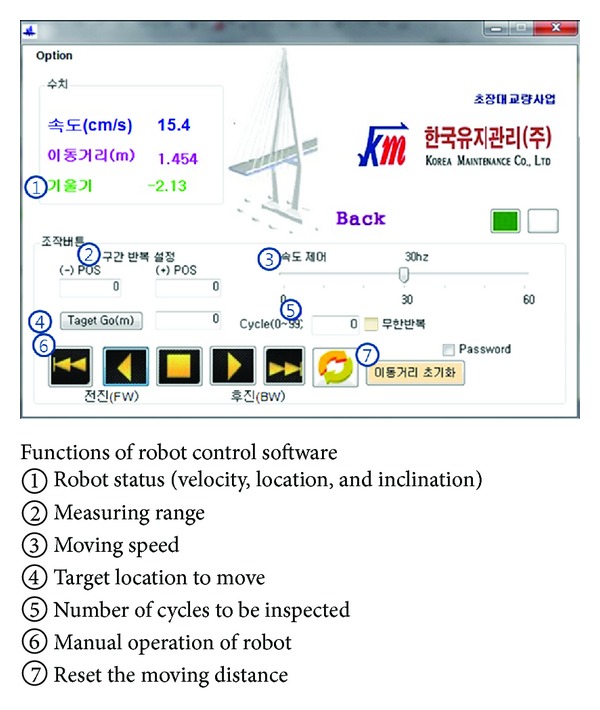
The climbing robot control software.

Three climbing mechanisms are usually employed for cable inspection robots, including magnetic, pneumatic, and electric methods [53, 54]. Electric method was chosen in this study for easy control and constant climbing force. Wireless communication is available between the climbing robot unit and the control and analysis subsystem to the robot control and sensor data transfer. The robot unit can be controlled by inspectors in distance of up to 600 m using a control software program shown in Figure 16.

#### 4.1.1. Cable-Suspension Bridge Robot

**Figure 17:**
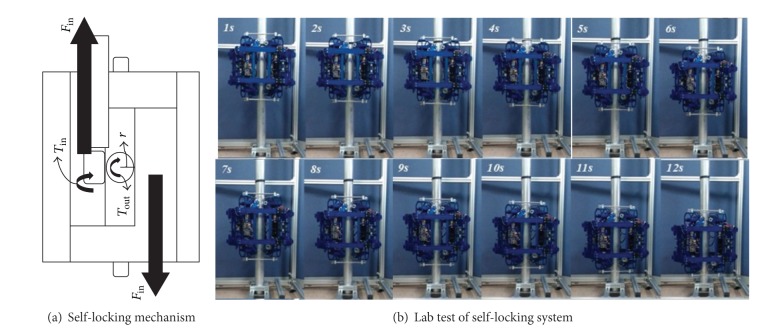
The climbing robot unit and self-locking mechanism for vertical hanger cables in suspension bridges [55].

An important design objective of the cable-suspension bridge robot was that the robot should be applicable to various gauge sizes of hanger cables. Another important design objective was that the robot should have enough climbing force to inspect vertical hanger cables, and, for unpredictable power outage, the gravity force due to the robot dead weight should be effectively counteracted to avoid freefall. To accomplish these design objectives, electrical DC motors are used to actuate the robot system on hanger cables. The robot system employed pantograph mechanism for various cables gauges and self-locking mechanism for power outage (see Figure 17). The self-locking system was designed to prevent reverse force on the motor and to reduce falling acceleration during power outage. A simple gear system is used, which consists of differential gears including worm and pinion gears and worm wheels attached to disk dampers [55, 56].

#### 4.1.2. Cable-Stayed Bridge Robot

**Figure 18:**
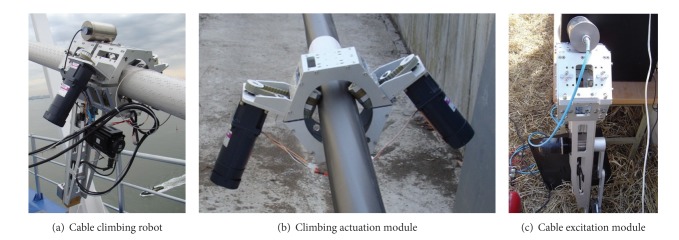
Cable climbing robot for stay cable inspection.

For cable-stayed bridges, the climbing robot subsystem was designed for two important design objectives. First, the robot should have enough climbing force to climb inclined stay cables. The other is that the climbing robot should be able to excite a stay cable to test cable dampers, whose dynamic response is separately measured with accelerometers installed on stay cables. Therefore, the actuation system of the climbing robot consists of two modules: (i) climbing actuation module and (ii) cable excitation module (see Figure 18(a)) [57]. The climbing actuation module has two electric motors to climb the robot unit on stay cables (see Figure 18(b)). The cable excitation module consists of a cable exciter associated with a pneumatic fixation device for the secure grip of the robot unit during cable vibration (see Figure 18(c)).

**Figure 19:**
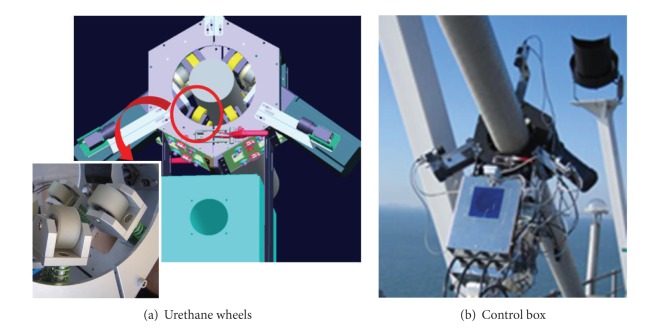
The cable climbing robot for stay cables in cable-stayed bridges.

Urethane wheels are used for fast movement on stay cables with improving friction and reducing cable surface damage during inspection (see Figure 19(a)). The wheel assembly consists of the wheels attached to springs and spacers inside the outer frame for adaptation to various cable gauges. A control box is located at the bottom of the robot for posture stability during movement (see Figure 19(b)).

### 4.2. Magnetic Sensing Module of NDT Subsystem

**Figure 20:**
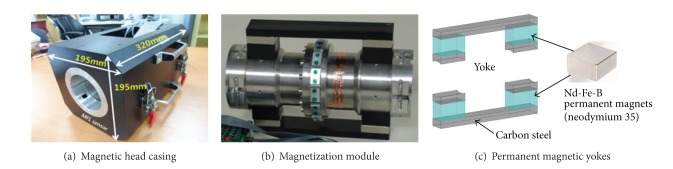
Magnetization module of the magnetic sensor.

The magnetic sensor head in the NDT subsystem consists of two modules, including (i) the magnetization module to generate a magnetic flux field and (ii) the sensing module to measure magnetic flux leakage caused by the presence of inner wire defects. The magnetic head is contained in an aluminum case with the dimensions of *W*  195 mm × *H*  195 mm × *L*  320 mm. The inner diameter of the casing is designed for steel cables with up to 85 mm diameter (see Figure 20(a)). The magnetization module generates a uniform magnetic field around a cable with a pair of yokes consisting of four high strength Nd-Fe-B permanent magnets (neodymium 35) and a plate of carbon steel (see Figures 20(b) and 20(c)).

**Figure 21:**
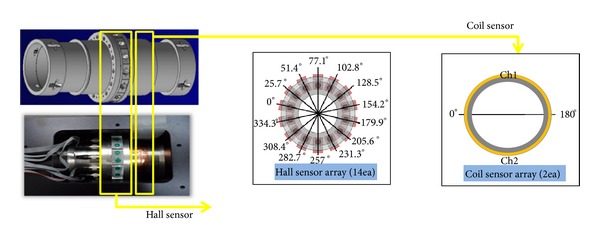
Sensing module of the magnetic sensor.

The sensing module has two types of sensors, including Hall effect sensors and coil sensors (see Figure 21). The Hall effect sensors have 14 channels arranged around a bridge cable to detect LF, such as inner wire breakage. The coil sensors have 2 channels to measure a total magnetic flux that is necessary information to calculate LMA. These sensors are connected to an on-board data acquisition system in the control box.

**Figure 22:**
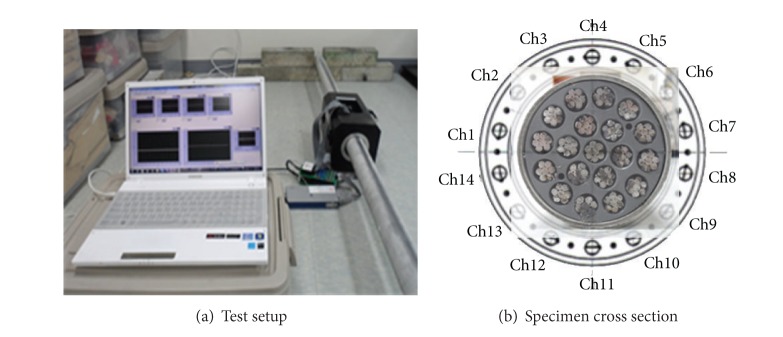
Laboratory tests of the magnetic sensor performance.

A series of laboratory tests were carried out to verify the magnetic sensor performance. Cable specimens with the diameter of 76 mm and length of 2 m had nineteen 7-wire strands contained in a PVC pipe (see Figure 22).

**Figure 23:**
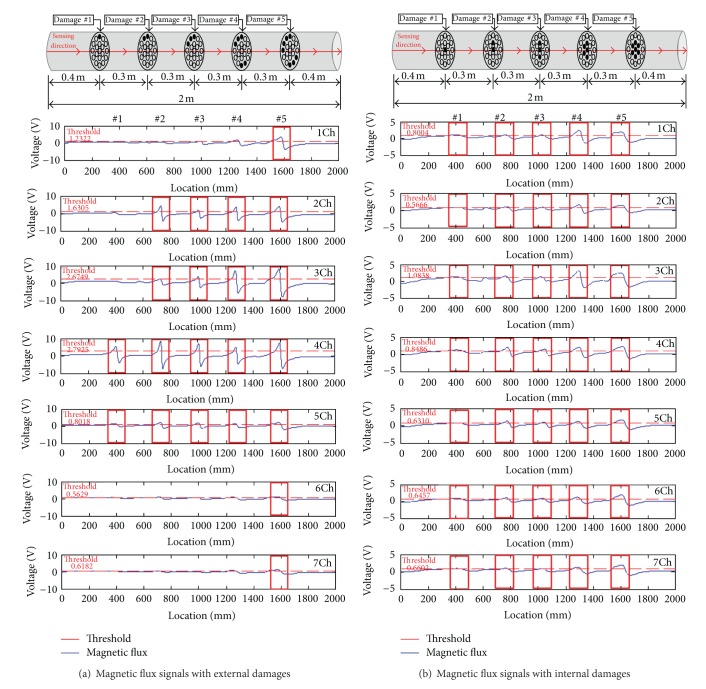
Test results of magnetic flux signals collected with the Hall effect sensors.

**Figure 24:**
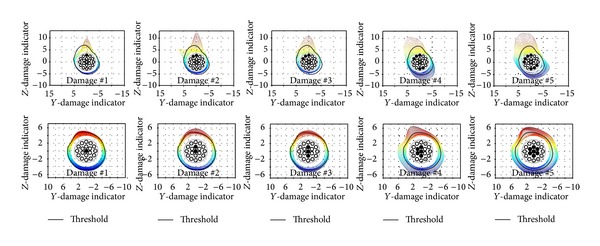
Visualized cross sections with threshold level.

Five damaged cases were tested by cutting strands at different locations to simulate LF.  Figure 23 shows magnetic flux signals measured using seven Hall effect sensors. The test result shows that the sensor magnitude is proportional to the proximity to damage location. Once the raw data were collected using the MFL sensors, the data were processed in the following procedures: (i) low pass filtering was applied to remove high frequency contents; (ii) a threshold was determined for the signal magnitude of Hall effect sensors through calibration using a laboratory mock-up test setup; (iii) LF locations were identified through visualization of contour mapping of the processed MFL data with respect to the threshold on the cable cross section (see Figure 24). Once the laboratory tests were conducted, the MFL sensor was applied to field tests on the Seohae Grand Bridge in Korea. The measured magnetic flux along the cables showed no significant fluctuation, and this means there is no abrupt change in the test cables.

### 4.3. Image Processing-Based Sensing Module of NDT Subsystem

**Figure 25:**
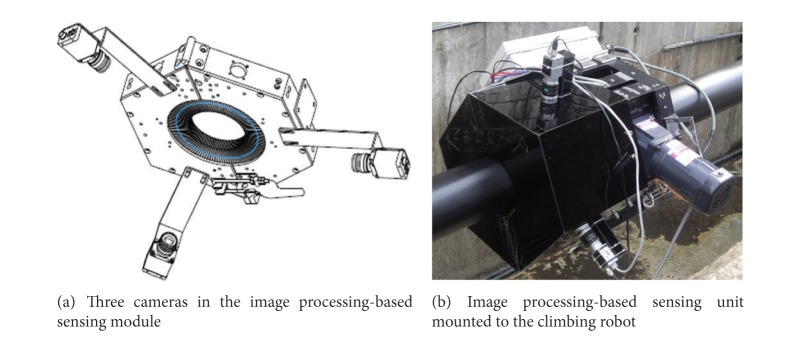
Image processing-based sensing module.

The image processing-based sensing module consists of three ruggedized cameras around a cable to collect digital images on cable surface (see Figure 25). The module collects raw cable surface images as the climbing robot moves on a bridge cable. The module has a high-speed wireless modem to transmit collected digital images to the server computer with the control and analysis subsystem.

**Figure 26:**
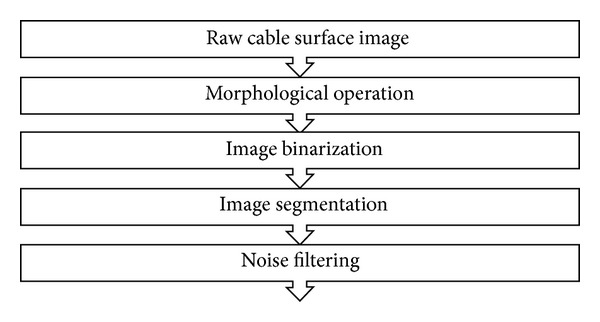
Morphological technique-based crack detection algorithm on cable surface.

A morphological technique-based image-processing algorithm was developed to detect crack-like defects on cable surface. The algorithm has several steps, including (i) morphological operation, (ii) image binarization, (iii) image segmentation, and (iv) noise filtering. The procedures of the image-processing algorithm are shown in Figure 26.

To detect crack-like cable surface defects, the morphological operation developed by Jahanshahi et al. was employed [58]:

(3) $$\begin{array}{c} T=\max\left[\left(I\circ S_{\left\{0^{{}^{\circ}},45^{{}^{\circ}},90^{{}^{\circ}},135^{{}^{\circ}}\right\}}\right)\cdot S_{\left\{0^{{}^{\circ}},45^{{}^{\circ}},90^{{}^{\circ}},135^{{}^{\circ}}\right\}},I\right]-I, \end{array}$$

where *I* is the original grayscale image of the cable surface; *S* is the structuring element that defines which neighboring pixels to be included in each morphological operation; ∘ is the morphological opening; · is the morphological closing; *T* is the grayscale processed image. To detect linear defects (i.e., cracks), a line pixel element was chosen as the structural element [58]. Since cracks on cable surface can have any orientation, the line-shape structuring element with four angles of 0°, 45°, 90°, and 135° was used during the morphological operation.

After the morphological operation, the output grayscale image was processed to binarize into black (i.e., cracks) and white (i.e., background). The Otsu threshold method was applied in the binarization process based on pixel interclass variance maximal [59].

After the binarization, the cable image is separated into crack pixels and background pixels. In this binarization image, however, no topological connectivity is established among the pixels. Thus, segmentation was conducted based on the connectivity of eight neighboring pixels of a crack pixel to determine which crack pixels belong to which cracks.

After the segmentation, noisy cracks were filtered out based on the three geometrical properties of segmented cracks, including crack area, maximum crack length, and crack eccentricity. Here, the crack eccentricity is 0 for a circle and 1 for a straight line.

**Figure 27:**
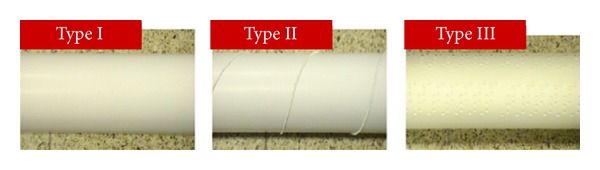
Three types of cables for lab tests: regular cable (type I), cable wound with a spiral wire (type II), and dimpled cable (type III) [16].

A series of laboratory tests were conducted using three types of bridge cables to evaluate applicability of the image-processing algorithm for various cable inspection conditions: regular cable (type I), cable wound with a spiral wire (type II), and dimpled cable (type III) (see Figure 27). Lines were marked with a black pen at various orientations to simulate crack-like defects on a mock-up test cable.

**Figure 28:**
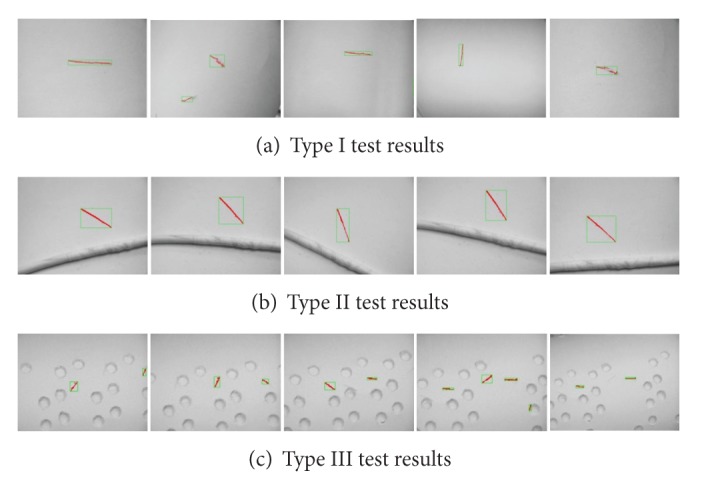
Image-processing test results to detect crack-like defects on different cable types.

The image-processing algorithm was applied to the test images. Crack-like marks on different cable types were accurately identified, and sample test results are shown in Figure 28. The detected defects are shown in red pixels in green boxes. The parameters used in the image processing-based crack detection algorithm are summarized in Table 3. Tests on the real bridge cables are being carried out currently.

## 5. Conclusion

This paper presented an overview on current bridge cable inspection practices and the cable inspection robot developed in Korea. Two types of robot systems have been developed for inclined cables in cable-stayed bridges and vertical hanger ropes in suspension bridges. The hardware of the cable inspection robots has the following unique features.

- The maximum cable diameters are 90 mm and 300 mm, and the maximum payloads are 25 kg and 45 kg for hanger ropes and stay cables, respectively.
- The robot is controlled with and transmits sensor data to the control system through wireless communication.
- Self-locking system is designed to prevent reverse force on the motor and dissipate freefalling force for unpredicted power outage.

The cable inspection robot system was tested in laboratory and field experiments to detect inner wire defects using a magnetic sensor and surface cable defects of bridge cables using an image processing-based sensing. The following results were observed from NDT tests.

- The magnetic sensors can be used to detect inner wire breakage by measuring magnetic flux change using the combination of two sensors, including 14-channel Hall effect sensors and two-channel coil sensors.
- The magnetic sensor module was packaged in a ruggedized aluminum case of *W*  195 mm × *H*  195 mm × *L*  320 mm. The total weight of the magnetic sensor was less than 20 kg.
- The image processing-based sensors can be used to detect crack-like surface defects with various orientations on cable surface. The crack detection algorithm was experimentally validated using three cable types, including regular cables, cables wound with spiral wires, and cable with dimples.

The robotic system is currently evaluated in realistic field conditions for robot mobility, defect detectability, and field applicability. Mobility is an important design concern for fast cable inspection with irregular surface conditions with obstacles on bridge cables. Detectability of various types of inner and outer defects on bridge cables should be validated under realistic field conditions. The bridge inspection robot should be applicable to various field conditions.

## Figures and Tables

**Table 1:** Bridge inspection standards in the United States and Korea. The table is modified from [6].

The United States	Korea
(i) AASHTO LRFD Bridge Design Specifications, 5th Ed. (2010)	(i) MOLIT^1^ Infrastructure Maintenance Guide (1995)
(ii) AASHTO Manual for Bridge Evaluation, Second Ed. (2011)	(ii) MOLIT (Bridge and Tunnel) Inspection and Diagnosis Guiding Principle of Details (1996)
(iii) AASHTO Guide Manual for Bridge Element Inspection, First Ed. (2011)	(iii) KISTEC^2^ The Bridge Inspection Handbook (1999)
(iv) AASHTO Guide for Commonly Recognized Structural Elements (1998)	(iv) MOLIT The Bridge Maintenance Manual (1999)
(v) AASHTO Manual for Condition Evaluation of Bridges (1994)	(v) MOLIT Concrete Structure Specifications (1999, 2003, 2007, and 2012)
(vi) AASHTO Manual for Maintenance Inspection of Bridges (1974, 1978, 1983, and 1993)	(vi) MOLIT Bridge Design Specifications (2000, 2005, 2008, 2010, and 2012)
(vii) AASHO Manual for Maintenance Inspection of Bridges (1970)	(vii) MOLIT Steel Structure Specifications (2003 and 2009)
(viii) FHWA Bridge Inspector's Reference Manual (2002 and 2006)	(viii) MOLIT The Bridge Maintenance Manual (2001)
(ix) FHWA, Bridge Inspector's Training Manual 90, 1991	(ix) MOLIT Maintenance Manual for Corrosion Protection of Steel Bridge (2003)
(x) FHWA Hydraulic Engineering Circular No. 18 (about 1988)	(x) KISTEC Manual for Bridge LCC Evaluation (2006)
(xi) FHWA “Scour at Bridges,” a technical advisory (1988)	(xi) MOLIT Bridge-Tunnel Inspection and Diagnosis (2007)
(xii) FHWA Inspection of Fracture Critical Bridge Members (1986)	(xii) KISTEC Know-how of Bridge Inspection: Ordinary Bridge and Cable Bridge (2008)
(xiii) FHWA Bridge Inspector's Training Manual 70 (1979)	(xiii) MOLIT Inspection and Diagnosis Guiding Principle of Details (2009)
(xiv) FHWA Culvert Inspection Manual (about 1979)	(xiv) MOLIT Inspection and Diagnosis Guiding Principle of Details Manual (2012)
(xv) FHWA The Bridge Inspector's Manual for Movable Bridges (1977)	
(xvi) FHWA Recording and Coding Guide for the Structure Inventory and Appraisal of the Nation's Bridges (1972, 1979, 1988, 1991, and 1995)	
(xvii) FHWA National Bridge Inspection Standards (1971, 1979, and 1988) (xviii) Code of Federal Regulations, 23 Highways Part 650, Subpart C—National Bridge Inspection Standards	

^1^MOLIT: Ministry of Land, Infrastructure and Transport. ^2^KISTEC: Korea Infrastructure Safety Corporation.

**Table 2:** Accelerometer-based cable monitoring systems in different countries [11].

Bridge	Nation	Main span (m)	Total number of cables (*a*)	Number of cables installed with accelerometer (*b*)	Ratio (*b*/*a*, %)
Seo-Hae	Republic of Korea	470	144	24	16.7
Jin-Do	Republic of Korea	344	60	18	30.0
Tatara	Japan	890	168	4	2.4
Jintang	China	620	168	20	11.9
Rion-Antirion	Greek	560	368	13	3.5
Oresund	Denmark	490	160	16	10.0
Fred Hartman	USA	381	192	19	9.9

**Table 3:** Parameters used in the image-processing tests.

Parameters	Type I	Type II	Type III
Structuring element shape	Line	Line	Line
Structuring element size	5 (pixels)	10 (pixels)	5 (pixels)
Structuring element orientation	[0° 45° 90° 135°]	[0° 45° 90° 135°]	[0° 45° 90° 135°]
Binary threshold	—	—	Otsu's filter
Crack connection (pixel)	20	20	20
Length threshold before connection (pixel)	40	70	—
Length threshold after connection (pixel)	60	70	—
Area threshold before connection (pixel)	30	100	10
Area threshold after connection (pixel)	40	100	—
Eccentricity threshold (pixel)	—	—	0.958
